# Dynamics of Radiation Damage Buildup in Ultrathin Hexagonal Boron Nitride Films under Ion Bombardment

**DOI:** 10.1021/acsami.5c25472

**Published:** 2026-05-04

**Authors:** Minsuk Seo, Leonardus Bimo Bayu Aji, Luis A. Zepeda-Ruiz, Sang Cheol Kim, Yan-Kai Tzeng, Steven Chu, Sergei O. Kucheyev

**Affiliations:** † 4578 Lawrence Livermore National Laboratory, Livermore, California 94550, United States; ‡ 6429 Stanford University, Stanford, California 94305, United States; § SLAC-Stanford Battery Research Center, SLAC National Accelerator Laboratory, Stanford, California 94025, United States

**Keywords:** 2D materials, hexagonal boron nitride, ion irradiation, radiation damage, defect dynamics, molecular dynamics

## Abstract

Two-dimensional hexagonal boron nitride (hBN) is attractive for several emerging applications. Ion bombardment can be used to modify the hBN properties. However, the understanding of radiation damage buildup in hBN remains limited. Here, we investigate the effects of the dose rate and ion mass on radiation damage buildup by studying 40 nm-thick hBN films bombarded at room temperature with 500 keV ^4^He, ^15^N, ^40^Ar, and ^129^Xe ions and comparing with results for ion bombardment of polycrystalline hBN ceramics. Raman spectroscopy is used to quantify damage buildup, and transmission electron microscopy is used for microstructural analysis. Experiments are complemented by molecular dynamics simulations of the formation and evolution of point defects. Lighter ions are found to be more efficient at disordering hBN than heavier ions. This observation points to a critical role of intracascade defect processes. In contrast, a negligible dose rate effect observed suggests limited intercascade defect dynamic annealing processes for these irradiation conditions. These findings provide a fundamental basis for hBN defect engineering.

## Graphical abstract

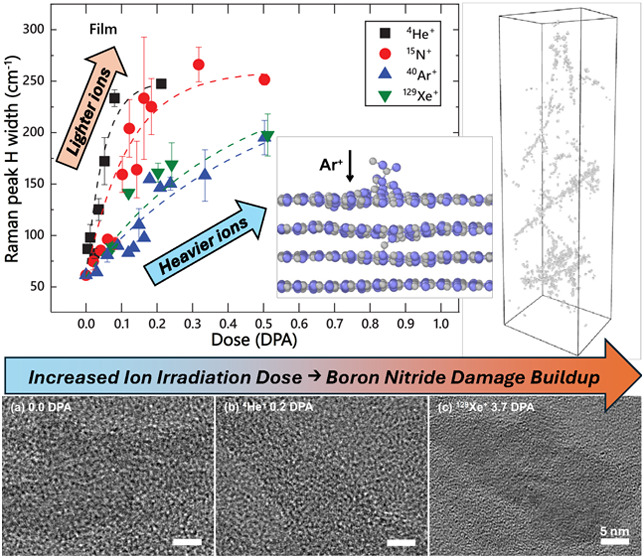

## I Introduction

Hexagonal boron nitride (hBN) is a refractory ceramic material with properties distinctly different from those of two related materials, pure diamond-like carbon and boron carbide. Similar to graphitic carbon, hBN is made of planar honeycombs of predominately covalently^1^ bonded B and N atoms stacked into layers held by weak van der Waals interactions.^2^ It has a unique combination of properties, including a high melting temperature of 2973 °C, a large band gap of ∼6 eV, a high strength of the B–N bonds (resulting in a Young’s modulus of ∼1 TPa), and a high thermal neutron absorption cross section of the ^10^B isotope. It is also chemically inert, resistant to oxidation in air to ∼1000 °C, and machinable. Because of these properties, hBN ceramics are used on the industrial scale in applications ranging from lubricants to cosmetics to nuclear reactor components.^3^

Two-dimensional hBN (2D-hBN) is an electrically insulating graphene-like material. Over the past decade, it has attracted large research interest^4^ owing to its emerging applications for electronics,^5^ biomaterials,^6^ quantum devices,^7–10^ radiation detectors,^11,12^ and energy-storage devices.^13^ Next-generation energy systems that would greatly benefit from hBN-based material innovations include Li-metal batteries, which have much higher theoretical capacities compared to conventional Li-ion batteries. Films of hBN could be used for interface stabilization and the suppression of dendrite growth. For example, similar to graphene membranes,^14^ hBN-based films could help mitigate Li dendrite formation in Li-metal batteries.^15–18^ Defects in hBN can promote Li interactions, highlighting the need for systematic studies of defect engineering in hBN.

Ion irradiation can be used to modify 2D-material properties for these functional applications.^9,10,19,20^ It could involve conventional semiconductor device fabrication steps such as ion implantation doping, dry etching, electrical isolation, and plasma-assisted film deposition when depositing atoms or ions crossing the plasma sheath at the substrate have sufficient energies for atomic displacement in the growing film.^21^ Radiation defect formation is the concomitant fundamental phenomenon for all of these ion-irradiation-based material processing techniques. Ion bombardment could also be used for so-called defect engineering for controlled modification of material properties via radiation-produced defects.^22–25^ For example, the mobility of Li ions through hBN membranes during electrochemical deposition depends on lattice defects that can be introduced by ion bombardment.^15,26,27^ This provides motivation to further investigate radiation defect engineering and its potential applications for hBN.

The formation of stable defects during ion bombardment is determined by both the ballistic phase of the collision cascade formation and the subsequent dynamic phase involving migration and interaction of point defects and occurring after thermalization of the collision cascade. Radiation dynamics determines how damage buildup depends on the dose rate, ion mass, and ion energy, as these irradiation parameters determine the displacement generation rate, collision cascade density, and degree of electronic excitation.

Despite several previous studies, our understanding of radiation damage in hBN is very limited compared to the case of other, more mature group-III semiconductors such as GaN, AlN, and InN.^28–30^ Several reports have demonstrated that hBN can be amorphized by high-dose ion bombardment at room temperature.^31–33^ A recent systematic study^33^ of damage buildup in 2D-hBN bombarded at room temperature with 500 keV Ar ions has revealed a monotonic damage buildup that can be described by the simplest zero-cascade overlap model.^34^ The extrapolation of these results^33^ for 500 keV Ar ion bombardment to other irradiation conditions, however, requires the understanding of the dynamic aspects of radiation damage buildup.

We are not aware of any previous systematic studies of the effects of the dose rate or collision cascade density for hBN. Although advanced methods of studying radiation defect dynamics involve pulsed ion beam bombardment,^29,35,36^ measurements of how radiation damage buildup depends on the dose rate and ion mass are typically the first step to study radiation defect dynamics. Here, we systematically study the damage buildup in ∼40 nm-thick hBN films bombarded at room temperature with 500 keV ^4^He, ^15^N, ^40^Ar, and ^129^Xe ions and compare with the case of hBN bulk ceramics irradiated under similar conditions. We also study the dose rate effect for hBN films irradiated with 500 keV Ar ions. Our results reveal that damage buildup is essentially independent of the dose rate and is strongly influenced by ion mass. Interestingly, in contrast to the case of cascade density effects in other semiconductors,^29^ hBN exhibits an “inverse” ion mass effect when lighter ions are more efficient at the formation of stable lattice defects. We attribute this to electronic excitation effects. Results of this study can be used for selecting irradiation conditions for specific applications.

## II Methods

All ion-irradiation experiments were performed with the 4-MV ion accelerator (model 4UH, National Electrostatics Corporation) at Lawrence Livermore National Laboratory with rastered ion beams. We used 10 × 10 × 1 mm^3^ sintered bulk polycrystalline hBN ceramic coupons (MTI Corp.) and ∼40 nm-thick multilayer hBN films grown on polycrystalline Cu foil by chemical vapor deposition (6Carbon Technology, China). Both films and bulk hBN ceramic samples were bombarded at room temperature with ion beams incident at 7° off the surface normal direction to minimize possible channeling effects. Table [Table tblI] summarizes the irradiation conditions of the present study. For films, the ion energy was 500 keV for all ion species since projected ion ranges, also listed in Table [Table tblI], were much larger than the film thickness. For bulk ceramic samples, ion beam energy was varied to limit the ion stopping range to ∼1 μm. Data for 500 keV Ar ions was taken from our recent study.^33^

**I tblI:** Summary of Ion-Irradiation Conditions of the Present Study[Table-fn tIfn1]

target	ion	energy (keV)	dose rate (10^12^ cm^–2^ s^–1^)	dose (10^12^ cm^–2^)	*R*_p_ (nm)
film	^4^He^+^	500	3.5	1000–90,000	1700
film	^15^N^+^	500	3.5	400–9500	886
film	^40^Ar^+^	500	1	80–1350	469
film	^129^Xe^+^	500	0.18	25–174	191
film	^40^Ar^+^	500	0.1	25–900	469
film	^40^Ar^+^	500	1	80–1350	469
film	^40^Ar^+^	500	2	120–710	469
film	^40^Ar^+^	500	5	25–900	469
bulk	^4^He^+^	200	3.5	600–29,000	922
bulk	^15^N^+^	500	3.5	100–9000	863
bulk	^40^Ar^+^	1000	1	5–1040	876
bulk	^129^Xe^+^	2500	0.18	7–143	902

a Also listed are projected ion ranges (*R*_p_) in bulk hBN estimated by TRIM code^37^ simulations.

Projected ion ranges and depth profiles of ballistically generated lattice vacancies were calculated with the TRIM code^37^ (version SRIM-2013.00, full cascade calculations, with Cu substrate recoils accounted in the case of hBN films) with an atomic concentration of hBN of 1.02 × 10^23^ atoms cm^–3^ (2.1 g/cm^3^) and the threshold energy for atomic displacements of 25 eV for both B and N sublattices.^33^ For both hBN films and bulk ceramics, the depth-averaged vacancy concentration was used for the calculation of ion doses in units of displacement per atom (DPA). The dose in DPAs was calculated by multiplying the vacancy generation function (in vacancies/cm/ion, from TRIM simulations), by ion dose (in ions/cm^2^), and dividing by the atomic density of hBN (in atoms/cm^3^). In the limiting case of low doses (i.e., without cascade overlap), the dose expressed in DPAs is the average volumetric density of ballistically generated vacancies. However, when collision cascades overlap, the number of atomic displacements is represented by the Poisson statistics rather than by the dose expressed in DPAs to account for some atoms, on average, being displaced more than once, while other atoms are not displaced at all. This reflects that during ion bombardment, defects are not introduced homogeneously into the material. Instead, they are introduced heterogeneously as discrete collision cascades with certain dimensions and (fractal) densities.^36,38^ Nevertheless, expressing doses in DPAs is a convenient and the most common way to compare cases with different irradiation conditions, and we do it here.

To study radiation damage in hBN films, we used the LAMMPS code^39^ to perform a series of molecular dynamics (MD) simulations designed to measure defect production and evolution in crystalline films bombarded with ions of different energy and mass. Interactions between B and N atoms were modeled with the Tersoff potential,^41^ and interactions between ions and B and N atoms in the film were described with a purely repulsive Ziegler–Biersack–Littmark (ZBL) potential.^37^ The hBN target was prepared by annealing a 10 × 10 × 50 nm^3^ film containing 471,040 atoms at 100 K with periodic boundary conditions along the *x* and *y* directions and a free surface at the top (and bottom) of the computational cell. Ion impact was along the *z* axis.

Due to the open nature of hBN, in MD simulations, the incident ions were directed onto the film perpendicular to the surface at random locations along nonchanneling directions. Otherwise, the ion would exhibit channeling and travel to the bottom surface of the simulation cell without producing any damage. We used He, N, Ne, and Ar ions with energies in the range of 100–500 keV. Since our MD simulations involved the interactions of different types of species that move at disparate velocities, we used a time step of Δ*t* = 10^–6^ ps. This time step size was determined such that the maximum particle displacement at each instant was Δ*r*_max_ = *r*_0_/30 = *V*_max_Δ*t*, where *V*_max_ is the velocity of the ion at 500 keV and *r*_0_ is the B–N interatomic distance in the layer. During the trajectory of the ion through the film, atoms were displaced from their lattice positions and created defects. These were identified and quantified with the OVITO visualization and analysis software.^40^

Film thickness was measured at the same 4-MV ion accelerator facility by Rutherford backscattering spectrometry with a 2 MeV ^4^He^+^ ion beam. Raman scattering measurements were performed in a backscattering configuration with 473 nm light and a Horiba Xplora microscope. The probing laser spot size was ∼2 μm. Spectra in a region of 800–2000 cm^–1^ were measured with a spectral resolution of 1 cm^–1^. The laser intensity was kept ≲2 mW to minimize sample heating. Peak parameters were extracted by fitting with a superposition of Gaussians, and the full width at half-maximum was used to quantify the peak width. The samples were measured three times at different locations, and the results were averaged with the standard deviation provided as error bars. The microstructure of films before and after ion irradiation was examined by transmission electron microscopy (TEM) in an FEI Titan TEM instrument operated at 300 kV.

## III Results and Discussion

Figure 1 shows representative Raman spectra from hBN films (Figure 1a,c) and bulk ceramic samples (Figure 1b,d) irradiated with either light ^4^He (Figure 1a,b) or heavy ^129^Xe (Figure 1c,d) ions to different doses. These spectra consist of three well-defined peaks, labeled C, H, and D. As discussed in detail in our previous study,^33^ these three peaks could be attributed to vibrations of atoms with *sp* ^3^ bonding characteristics of cubic BN (cBN, peak C at ∼1270 cm^–1^), the *E*_2*g*_-symmetry mode of sp^2^ bonded hBN (peak H at ∼1370 cm^–1^), and the bonding in disordered hBN regions (peak D at ∼1600 cm^–1^).^42–45^ The width of peak H serves as a useful measure of the structural quality of hBN.^45^ The disorder-related peak D has been attributed to the *E*_1*u*_-symmetry longitudinal optical mode, which becomes Raman-active when lattice symmetry is disrupted by disorder.^44^

**Figure 1:**
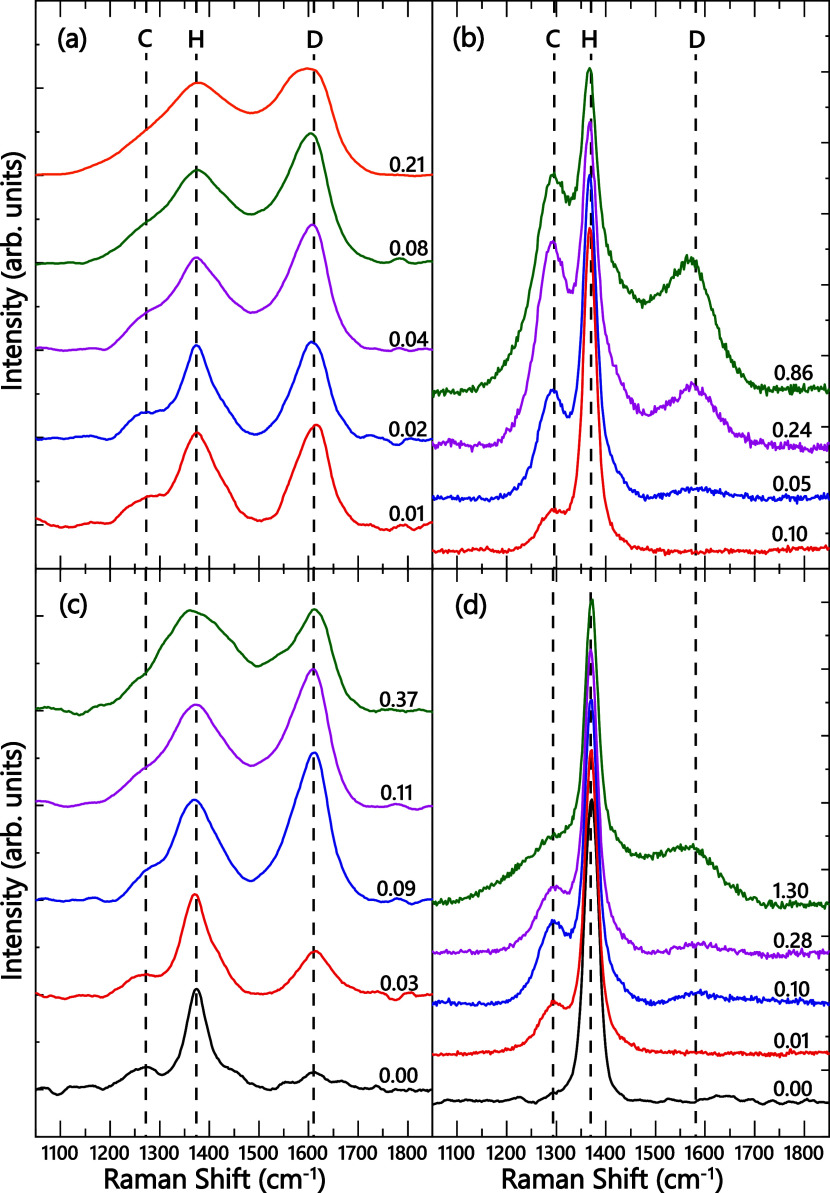
Representative Raman spectra from (a, c) 2D-hBN films and (b, d) bulk BN samples after different doses, shown in units of DPA, of bombardment with (a, b) ^4^He and (c, d) ^129^Xe ions. Vertical dashed lines mark peaks, labeled C, H, and D, assigned to vibrations of cubic, hexagonal, and disordered bonding configurations of BN, respectively.

Before ion irradiation, the Raman spectrum from a ceramic sample in Figure 1d exhibits a single H peak with C and D peaks being absent. The spectrum from the unirradiated film in Figure 1c contains all three peaks (H, C, and D). Irradiation of both film and bulk samples results in the broadening of the hBN-related peak H and an increase in the intensity of the disorder-related peak D. For bulk ceramic samples, peaks C and D appear at doses of 0.01 and 0.05 DPA, respectively, and their intensity increases with increasing dose.

Radiation damage buildup in hBN can be quantified by the analysis of the width of peak H.^33^ Figure 2 shows the ion dose dependence of the width of peak H for different ion species for cases of hBN films (Figure 2a) and bulk samples (Figure 2b). It reveals that, before ion irradiation, widths of peak H in films and bulk ceramics are ∼60 and ∼27 cm^–1^, respectively. The H peak width monotonically increases with increasing ion dose for all ion species for both film and bulk samples. The damage buildup follows a well-known phenomenological zero-cascade overlap model (the Poisson distribution of ion impacts)^34^ for all ion species, shown by dashed lines in Figure 2. Peak width, however, asymptotes to different values for light (He and N) and heavy (Ar and Xe) ions for the case of bulk ceramic samples (Figure 2b).

**Figure 2:**
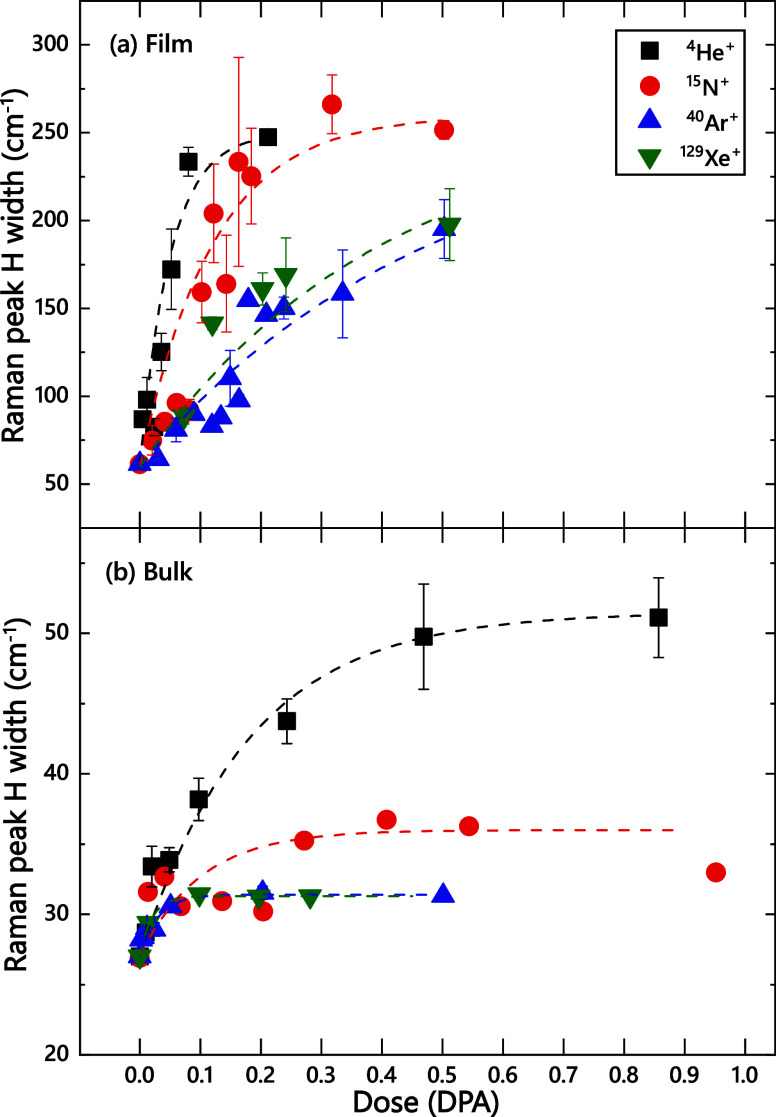
Ion dose dependencies of the width of the main Raman peak labeled H in Figure 1 for (a) 2D-hBN films and (b) bulk BN samples. Dashed lines represent the fitting with the zero-cascade-overlap damage build-up model. The legend in panel (a) applies to both panels.

There are also significant differences in the damage build-up behavior between bulk and film samples in Figures 1 and 2. For example, for He ion irradiation, damage buildup is faster for films than for bulk ceramics, with damage level saturation occurring at doses of ≳0.2 and 1 DPA, respectively. The main difference in the damage buildup for films and bulk samples is, however, in the saturation level of the width of the Raman peak H. It is ∼30–50 cm^–1^ for bulk and ≳200 cm^–1^ for films. Such a difference in the saturation level can be attributed to differences in the Raman probing depth for these two configurations of a thin film on a metallic substrate and a bulk ceramic sample. The laser light used in these experiments has a photon energy (2.6 eV) that is much lower than the band gap of hBN (∼6 eV). Hence, while the Raman signal from thin hBN films on Cu substrates represents depth-averaged film properties, the Raman signal from bulk ceramic samples is a superposition of scattering signals from the near-surface layer damaged by ion irradiation and the unirradiated bulk material. This leads to an overall reduction in the width of peak H. The minimum Raman probing depth for these experiments is defined by (both focusing and detection) optics of the instrument and can be estimated^46^ as ∼1.4*n*λ/(*NA*)^2^ ≈ 2 μm, where λ is the light wavelength (473 nm), *n* is the hBN refractive index at this λ (2.3),^47^ and NA is the lens numerical aperture (0.9).

To better illustrate the effects of ion irradiation on sp^3^ bonding in hBN, Figure 3 shows ion dose dependencies of the intensity ratio (C/H) of Raman peaks C and H in films (Figure 3a) and bulk (Figure 3b) samples. Before irradiation, the C/H ratio is zero for bulk samples (because peak C is absent) and 0.39 ± 0.12 for films, which could be attributed to the presence of sp^3^ bonding due to a high plasma potential and associated energetic ion bombardment during the chemical vapor deposition of these films. For bulk samples (Figure 3b), ion irradiation results in the appearance of peak C and a monotonic increase in the C/H ratio up to a saturation value, which depends on ion mass. For doses of ≳0.3 DPA, irradiation of bulk samples with lighter He and N ions results in larger C/H ratios compared to cases of Ar and Xe irradiation.

**Figure 3:**
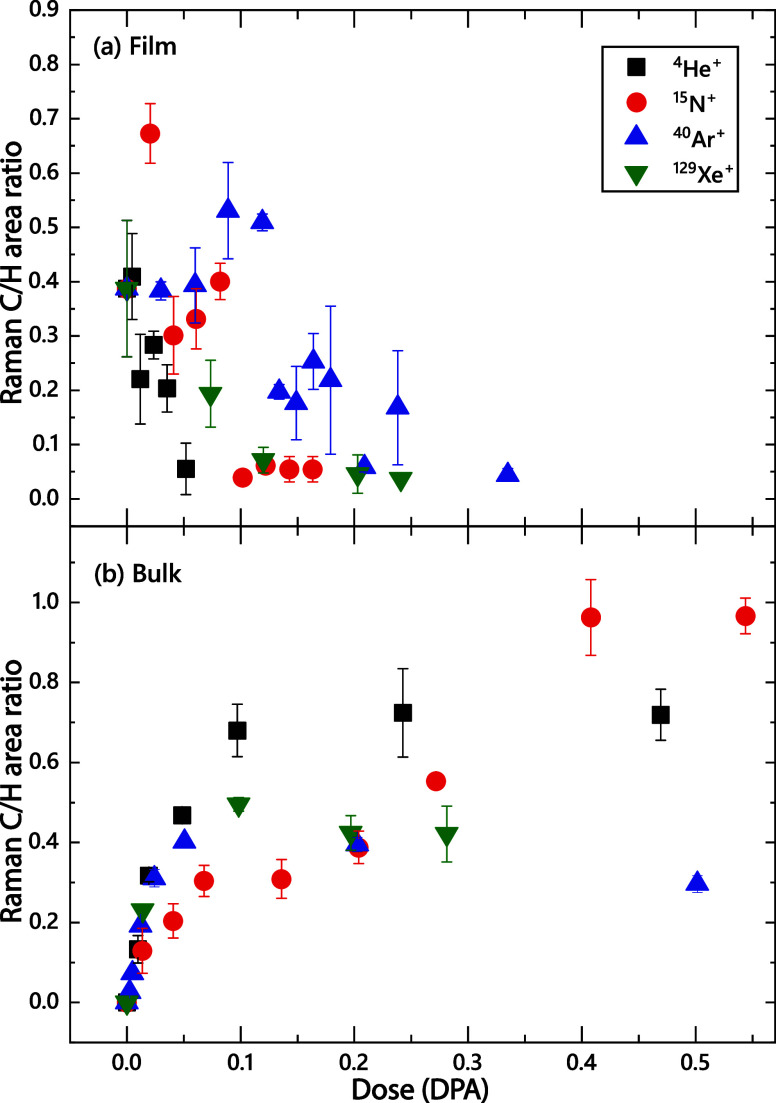
Ion dose dependencies of intensity ratios of the main peaks labeled C and H in Figure 1 for (a) 2D-hBN films and (b) bulk BN samples. The legend in panel (a) applies to both panels.

For films (Figure 3a), the behavior of the C/H peak intensity ratio is qualitatively different and more complex. For He and Xe ions, it monotonically decreases with increasing ion dose. The damage buildup is accompanied by an elimination of regions with sp^3^ bonding. For N and Ar ions, a nonmonotonic dependence is observed, when the C/H intensity ratio peaks at ∼0.1 DPA, followed by a reduction for larger doses. The bombardment with N ions leads to the highest C/H ratio of ∼0.67 at a relatively low dose of 0.02 DPA. This difference in the behavior of the C/H intensity ratio for film and bulk samples could be attributed to effects of the sample surface as well as the substrate/film and cBN/hBN interfaces in such thin films with a relatively large surface-to-volume atomic ratio. The BN surface is hBN-rich even for predominantly cBN films.^52^ Irradiation-induced *sp* ^3^-to-*sp* ^2^ bonding transitions could originate at such interfaces and be aided by lattice defects.^53^ Such an influence of interfaces, including the free surface and boundaries between different phases and different materials, on radiation damage is expected based on previous extensive studies for different material systems, both metals and ceramics,^48,50,51^ including another nitride, GaN.^49^ Interfaces can act as defect traps or annihilation sites, and they change the defect accumulation in regions defined by effective defect diffusion lengths.

The influence of the ion mass on the defect microstructure in hBN is illustrated by Figure 4, which shows bright-field TEM micrographs of BN films before and after bombardment with He or Xe ions to different doses. Prior to ion irradiation (Figure 4a), the film is composed of ≲100 nm-wide hBN crystallites and regions of amorphous BN. The crystallites exhibit two different *d*-spacings, 0.33 and 0.24 nm, which correspond to the (0002) and (1100) planes of hBN, respectively.^54,55^ There is no evidence of turbostratic BN (tBN, which is a phase when layers are stacked with rotations or translations relative to one another)^55,56^ in films either before or after irradiation. There is also no evidence of cBN crystallites in the films. This suggests that Raman peak C is associated with amorphous regions of *sp* ^3^-bonded BN rather than with crystalline cBN phase inclusions.^56^ Irradiation with He ions to a dose of 0.2 DPA amorphizes the BN film, and crystallites are no longer observed, as shown in Figure 4b. Lattice amorphization of hBN films after a dose of 0.64 DPA of 500 keV ^40^Ar ions has also been observed by TEM in our previous study.^33^ In contrast, after a much larger dose of 3.7 DPA of heavy ^129^Xe ions, small hBN crystallites still remain in the film (Figure 4c). The width of the Raman peak H is 178 ± 23 cm^–1^ for this film. Hence, films could still exhibit partial crystallinity, even when Raman spectra feature a broad H peak.

**Figure 4:**
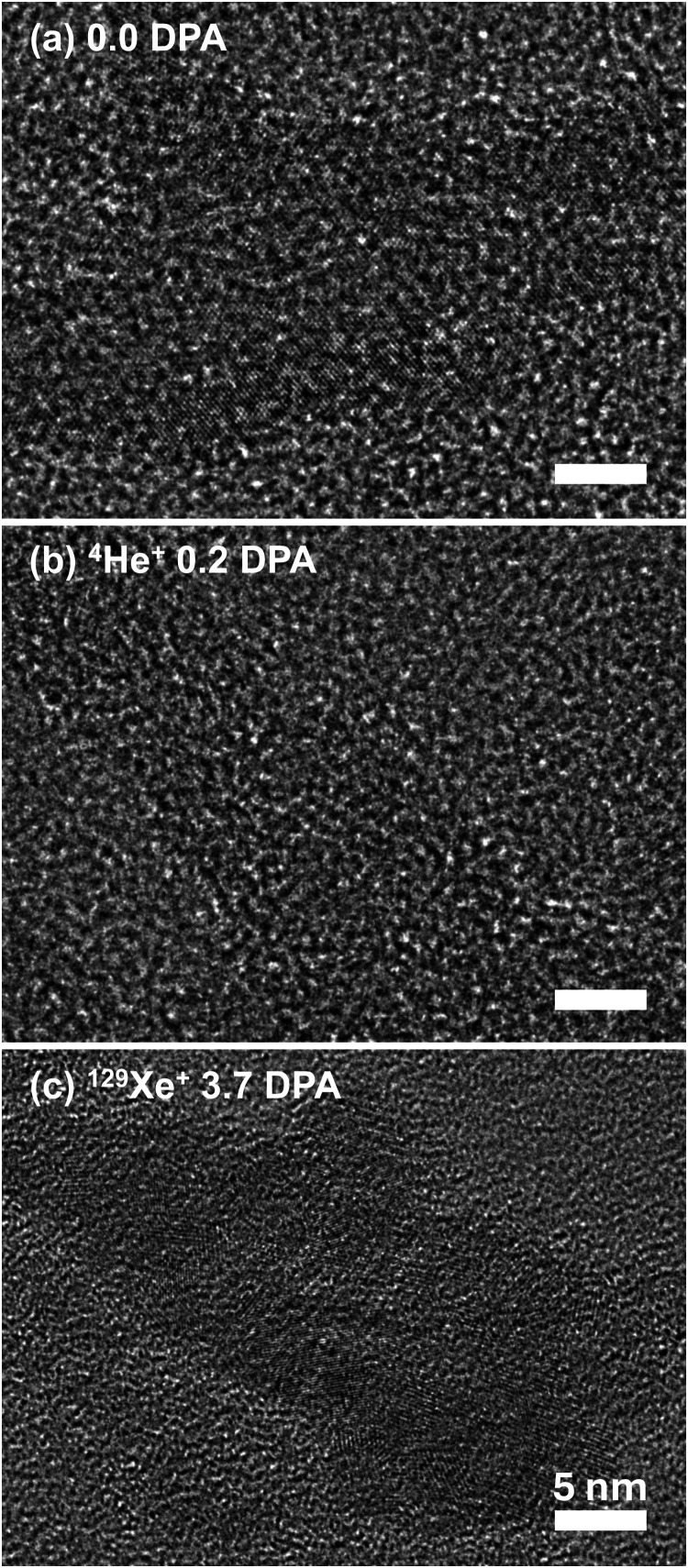
Selected TEM micrographs of (a) an as-grown hBN film and films irradiated at room temperature with (b) 500 keV He ions to a dose of 0.2 DPA and (c) 500 keV Xe ions to a dose of 3.7 DPA. All scale bars are 5 nm.

Ion bombardment of hBN leads to atomic displacements and the creation of point defects when target atoms get displaced from their equilibrium position. The simplest point defects are so-called Frenkel pairs, which are isolated vacancies and interstitials. Due to its layered structure, hBN could exhibit point defect structures that are more complex than the simple Frenkel pair components and their clusters. A small subset of commonly observed defects in layered materials such as hBN is depicted in Figure 5, showing results of our MD simulations illustrating a 10-atom ring in the basal plane (Figure 5a), dangling bonds, and interlayer atoms (Figure 5b).

**Figure 5:**
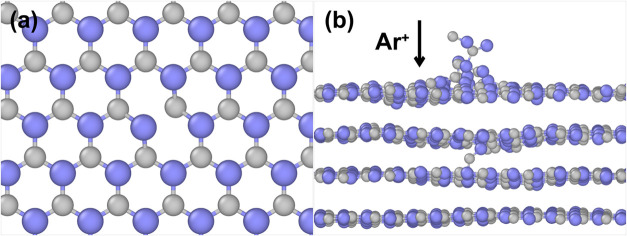
Results of MD simulations illustrating (a) 10-atom rings and (b) dangling bonds and interlayer atoms produced by ion bombardment of multilayer hBN. Smaller (gray) and larger (purple) balls represent N and B atoms, respectively.

Figure 6 shows predictions of MD simulations for the evolution of defects during and after their ballistic generation by either a 100 keV Ar ion (Figure 6a,b) or 500 keV ions with different masses (Figure 6c). It is seen from Figure 6c that the number of defects close to linearly increases with time as ions propagate through the film through a collision cascade, displacing atoms in different layers. The ballistic phase of the cascade completes at ∼0.2 ps when the ion has penetrated the film, and it is at rest. After that, defects experience relaxation with an ∼30% reduction in their concentration during the 3 ps relaxation time interval shown in Figure 6c. Defect annihilation is limited for hBN, in contrast to predictions of MD simulations for metallic material systems, for which only a fraction of ballistically generated point defects survive the thermalization phase of the collision cascade at room temperature.^57^ This illustrates a critical role of atomic bonding in radiation defect accumulation, complexity of the physics of radiation defects, and challenges in translating research findings from one material system to another.

**Figure 6:**
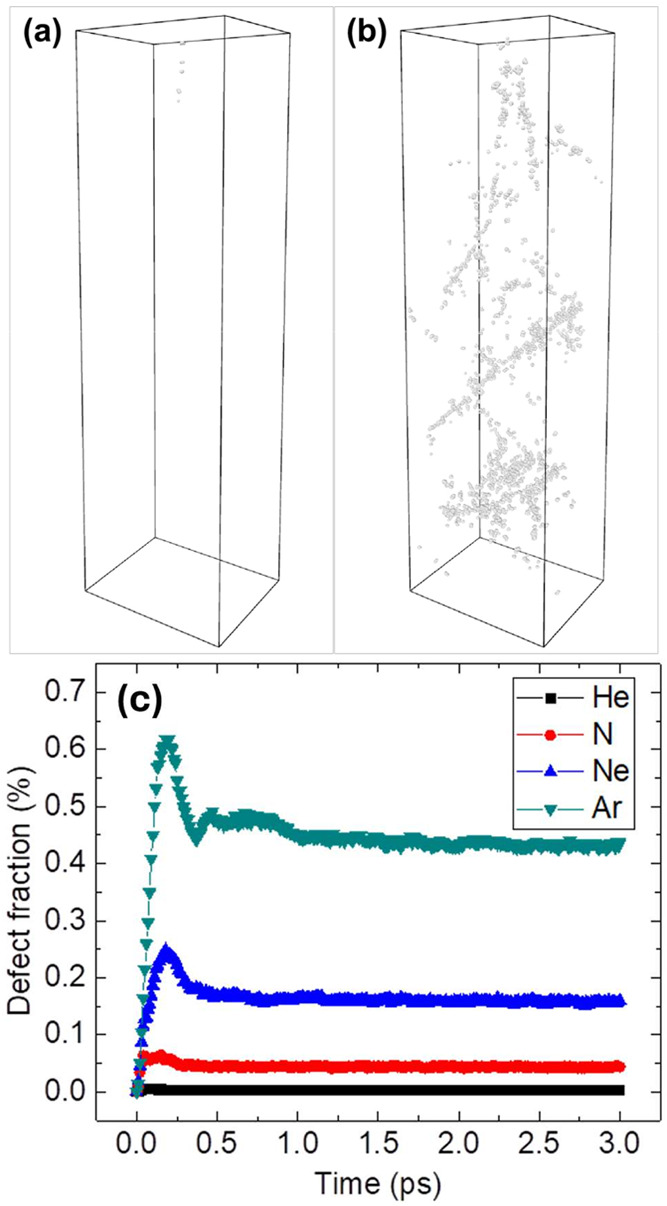
Typical result of MD simulations showing defects produced by a 100 keV Ar ion in a 40 × 35 × 125 nm^3^ computational cell containing 16,384,000 atoms. For clarity, defects are shown only for time intervals of (a) 0.2 ps and (b) 2.5 ps after the Ar ion enters the surface. (c) Time evolution of the atomic fraction of defects in the simulation cell for 500 keV ions with different masses.

Figure 7 shows predictions of MD simulations of the dependence of the defect density after the cascade thermalization (at 3 ps) on the ion energy for ions with different masses. Large errors reflect the limited statistics since only 5 ion impacts were simulated for each irradiation condition. Nevertheless, Figure 7 shows that heavier ions create more defects (per ion) and that the defect concentration decreases with increasing ion energy, which could be attributed to a corresponding reduction in the nuclear stopping power.

**Figure 7:**
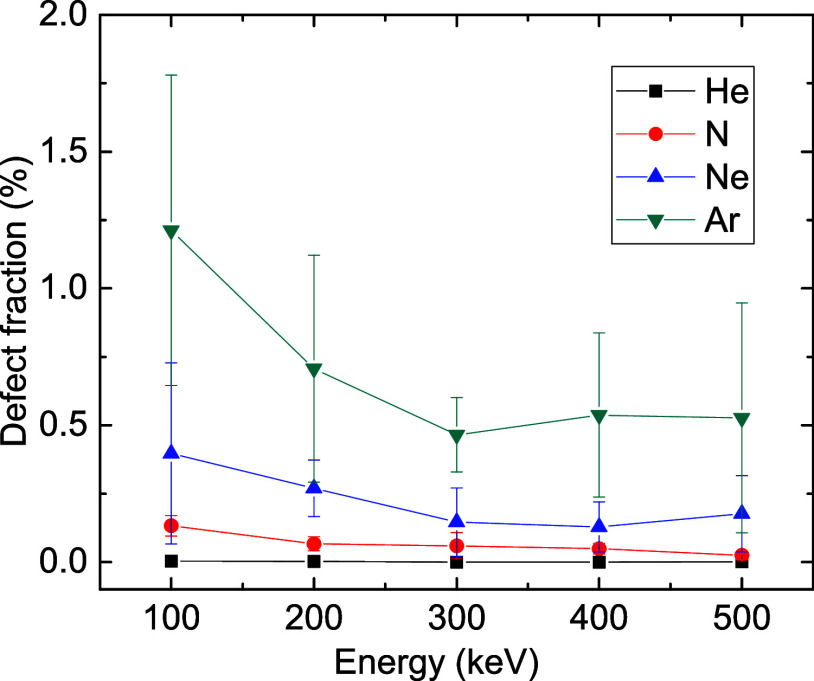
Dependence of the atomic fraction of defects in the MD simulation cell at 3 ps on ion energy for ions with different masses.

The above results clearly show that ion mass plays a key role in damage accumulation in hBN under ion irradiation. Based on studies of radiation damage processes in other semiconductors, including Si and other group-III nitrides such as GaN, AlN, and InN, heavier ions are expected to be more efficient in the formation of stable lattice disorder than lighter ions.^28–30,58^ This is related to the collision cascade density, which generally scales with ion mass.^29,36,38,59,60^ Mobile (and hence unstable during ion irradiation) point defects in denser cascades experience more efficient clustering to stable defect structures that are characterized after ion irradiation by, for example, Raman spectroscopy as in the present study.

In contrast to such expectations, for hBN, lighter ions (^4^He and ^15^N) cause more efficient damage accumulation than heavier ions (Figures 1 and 2). We attribute this to the effects of electronic interactions. Indeed, energetic ions interact with target atoms via so-called electronic and nuclear energy loss processes.^29^ Electronic energy loss (*E*_e_) comes from the Coulomb interaction of (screened by bound electrons) nuclei of ions with target atom electrons. It leads to the electron excitation and ionization. Nuclear energy loss (*E*_n_) involves Coulomb interaction of the nuclei of ions and target atoms, leading to atomic displacements when the energy transferred is above the threshold energy for atomic displacements.

Table [Table tblII] shows that, for the ion energy studied here, *E*_*e*_ increases with increasing ion mass from He to N and remains almost constant for heavier ions. In contrast, *E*_*n*_ monotonically increases with the mass in the entire mass range. As a result, the *E*_e_/*E*_n_ ratio decreases with an increase in ion mass. Relaxation processes after intense electronic excitation could lead to atomic displacements and defect formation via either bond destabilization or collective processes of thermal spikes and/or Coulomb explosion.^61–63^ This is not the case here, since both *E*_e_ and the combined energy loss values (*E*_e_ + *E*_n_) increase with increasing ion mass (Table [Table tblII]), while the efficiency of the formation of stable damage decreases (Figure 2). Electronic interactions could also facilitate the formation of lattice defects.^64^ This is again in contrast to our experimental observations. In some ceramics, electronic excitation could lead to defect annealing, resulting in lower damage levels for irradiation conditions with larger *S*_e_/*S*_n_ ratios.^65^ This is again in contrast to our observations of more efficient damage accumulation for irradiation conditions with larger *S*_e_/*S*_n_. Instead, our results suggest that electronic excitation facilitates the clustering of the point defects. Mechanisms of such an increased defect formation could, for example, involve an increased defect mobility and/or lowered energy barriers for defect clustering processes for irradiation conditions with larger *E*_e_/*E*_n_ ratios. More work, involving atomistic modeling, is needed to identify the atomic-scale processes behind these intriguing observations.

**II tblII:** Electronic (*E*_e_) and Nuclear (*E*_n_) Energy Loss Values for 500 keV Ions in hBN[Table-fn tIIfn1]

ion	*E*_e_ (eV/Å)	*E*_n_ (eV/Å)	*E*_e_/*E*_n_
^4^He^+^	41	0.1	554
^15^N^+^	73	2.2	34
^40^Ar^+^	86	24	3.6
^129^Xe^+^	78	197	0.4

a Results of TRIM code^37^ simulations.

Finally, Figure 8 plots ion dose dependencies of the width of Raman peak H for films bombarded with 500 keV Ar ions with different dose rates in a wide range of (1–50) × 10^11^ cm^–2^ s^–1^. It reveals a negligible dose rate effect: within experimental errors, the damage buildup is independent of the dose rate. A dose rate effect originates from the interaction of (mobile) point defects generated in adjacent collision cascades.^29,66,67^ Depending on which processes dominate the damage buildup, the interaction of point defects could lead to either their annihilation (and a reduction in the concentration of stable defects) or defect clustering, leading to higher defect densities.^29,66–68^ The negligible dose rate effect revealed by Figure 8 indicates a limited role of dynamic intercascade defect interaction processes in the formation of stable damage in hBN for these irradiation conditions.

**Figure 8:**
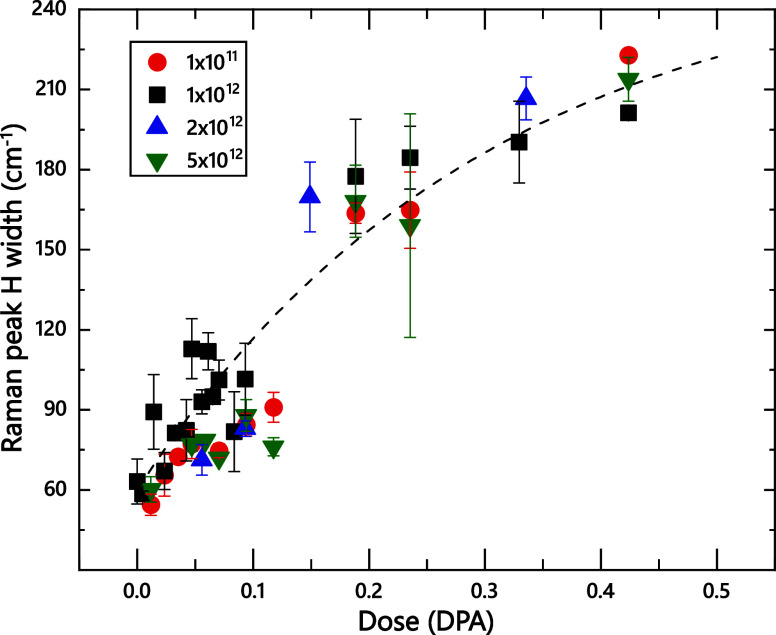
Ion dose dependencies of the width of the main Raman peak labeled H in Figure 1 for 2D-hBN films bombarded with 500 keV Ar ions with different dose rates, indicated in the legend (in units of cm^–2^ s^–1^). The dashed line is the fitting with the zero-cascade-overlap damage build-up model.

## IV Summary

We have investigated the damage buildup in ∼40 nm-thick 2D-hBN films and polycrystalline bulk hBN samples irradiated at room temperature with ^4^He, ^15^N, ^40^Ar, or ^129^Xe ions under conditions listed in Table [Table tblI]. Our main results can be summarized as follows.

- The layered structure of hBN adds complexity to the types of radiation-generated point defects.
- Damage buildup follows the simplest zero-cascade overlap behavior for all ion masses for both films and bulk samples.
- There is a pronounced mass effect on damage buildup. Lighter ions exhibit a higher efficiency in the formation of stable lattice defects than heavier ions. This has been attributed to intracascade effects of electronic excitation that could involve an increased defect mobility and/or lowered energy barriers for defect clustering processes for irradiation conditions with lighter ions.
- The absence of a dose rate effect for 500 keV Ar ions suggests a negligible role of intercascade defect interaction processes for these irradiation conditions.
